# Functional Differentiation of Antiporter-Like Polypeptides in Complex I; a Site-Directed Mutagenesis Study of Residues Conserved in MrpA and NuoL but Not in MrpD, NuoM, and NuoN

**DOI:** 10.1371/journal.pone.0158972

**Published:** 2016-07-08

**Authors:** Eva Sperling, Kamil Górecki, Torbjörn Drakenberg, Cecilia Hägerhäll

**Affiliations:** 1 Department of Biochemistry and Structural Biology, Center for Molecular Protein Science, Lund University, Lund, Sweden; 2 Department of Biophysical Chemistry, Center for Molecular Protein Science, Lund University, Lund, Sweden; University of Saskatchewan, CANADA

## Abstract

It has long been known that the three largest subunits in the membrane domain (NuoL, NuoM and NuoN) of complex I are homologous to each other, as well as to two subunits (MrpA and MrpD) from a Na^+^/H^+^ antiporter, Mrp. MrpA and NuoL are more similar to each other and the same is true for MrpD and NuoN. This suggests a functional differentiation which was proven experimentally in a deletion strain model system, where NuoL could restore the loss of MrpA, but not that of MrpD and vice versa. The simplest explanation for these observations was that the MrpA and MrpD proteins are not antiporters, but rather single subunit ion channels that together form an antiporter. In this work our focus was on a set of amino acid residues in helix VIII, which are only conserved in NuoL and MrpA (but not in any of the other antiporter-like subunits.) and to compare their effect on the function of these two proteins. By combining complementation studies in *B*. *subtilis* and ^23^Na-NMR, response of mutants to high sodium levels were tested. All of the mutants were able to cope with high salt levels; however, all but one mutation (M258I/M225I) showed differences in the efficiency of cell growth and sodium efflux. Our findings showed that, although very similar in sequence, NuoL and MrpA seem to differ on the functional level. Nonetheless the studied mutations gave rise to interesting phenotypes which are of interest in complex I research.

## Introduction

NADH:quinone oxidoreductase (complex I) is the largest enzyme complex in the respiratory chain. In comparison to the other complexes in the chain, a complete high resolution structure became available very late, which shows how challenging this huge enzyme complex is [1–5]. The classical bacterial complex I is composed of 14 different protein subunits [6, 7], seven of which form the hydrophilic, cytoplasmic domain and the other seven the hydrophobic, membrane-embedded domain. The electron transfer through the promontory part of the enzyme, i.e. from the NADH binding site via a series of FeS clusters to the quinone binding site [8], is coupled to proton pumping across the membrane-spanning domain. Under most conditions the stoichiometry is 4H^+^/2e^-^ [9–11]. However, the actual coupling mechanism between the two activities remains poorly understood.

The membrane domain of complex I contains seven subunits. The three largest of those, NuoL, NuoM, and NuoN, which are very similar to each other [12] and also share homology to a Na^+^/H^+^ antiporter, Mrp, (multiple resistance and pH) [13]. This protein complex exists in Gram-positive bacteria, and enables growth in high salt concentrations and high pH. Due to this homology, the antiporter-like subunits were suggested as prime candidates for conducting proton translocation in complex I. The Mrp complex consists of seven proteins, two of which, MrpA and MrpD, are homologous to each other [14]. In addition, it was noticed that the five antiporter-like polypeptides (MrpA and D, NuoL, M, and N) from various organisms formed two distinct phylogenetic clusters, and that the complex I subunit NuoL consistently grouped with MrpA, whereas MrpD was more similar to NuoM and NuoN [15]. This difference must therefore have been preserved throughout evolution, and it suggests a somewhat different function of the MrpA-like and MrpD-like polypeptides. As demonstrated in our previous work, the disparity between the polypeptides is indeed also functional [16, 17]. Deletion of either *mrpA* or *mrpD* genes from *Bacillus subtilis* resulted in a Na^+^ and pH sensitive growth phenotype (*ΔmrpA* cells were unable to grow in 80 mM NaCl, and *ΔmrpD* cells in 60 mM NaCl [17]). Expression of the deleted proteins from a plasmid rescued the growth, but MrpA could never compensate for the loss of MrpD and *vice versa* in the deletion strains. Interestingly, expression of NuoN could under some circumstances rescue the growth of the *B*. *subtilis* Δ*mrpD* strain and NuoL could likewise restore the loss of MrpA, but not the other way round. Taken together, the simplest explanation for these results was that each polypeptide is not an antiporter, but MrpA and MrpD are single ion channels that together form a Na^+^/H^+^ antiporter. Even if the function of NuoM remained experimentally undetermined [17], we concluded that complex I seem to contain one Na^+^ channel and two H^+^ channels.

Many residues are conserved in the entire family of these proteins, and the three homologous complex I subunits show an almost perfect structural overlap [1, 4]. Corresponding mutations have often showed reduced activity, but this was interpreted as NuoL, NuoM and NuoN having a common role in complex I [18]. A detailed list of all mutants available so far and a summary of their properties in NuoLMN is compiled in the supplementary material of [4]. Mutations with the most drastic effects on complex I are listed in [19]. An extensive mutagenesis study in MrpA is reported in [20].

In the present work, we investigated further the relationship between NuoL and MrpA, which we believe are Na^+^ channels. As a starting point we used a sequence alignment between the different Mrp and antiporter-like complex I subunits. We searched for residues that are only conserved in NuoL and MrpA, but not in the other subunits. We could identify several residues, but found a particular stretch of four residues in the transmembrane helix VIII. This helix lies in a direct proximity to the broken helix VII in NuoL. Since broken helices are vital parts of ion translocation machineries [21], we considered this position promising to study the role in MrpA and NuoL function. Also, two residues conserved in all five antiporter subunits were chosen for mutation, as they were previously shown to drastically decrease complex I activity [22]. We studied the effects of the mutations in the *B*. *subtilis* complementation assays with a deletion strain that lacks the gene for *mrpA*, which leads to a salt sensitive phenotype. We then expressed *in trans* different NuoL and MrpA mutants, which are all fused to a cytochrome c domain including a his-tag and recorded their growth abilities over time. In addition to studying the cell growth, the sodium conduction in the deletion strain expressing the mutant proteins was measured by ^23^Na-NMR method earlier developed for Gram-positive bacteria [23].

The results of our study show that NuoL and MrpA differ on the functional level, despite their high sequence similarity. Only one of the four mutations (M225I/M258I) showed a similar phenotype in both proteins and therefore seems to be important in both proteins. We also demonstrate that using complementation studies in combination with ^23^Na-NMR is a useful way to pinpoint and study new mutations in complex I and Mrp.

## Materials and Methods

### General molecular biology

Bacterial strains, plasmids and primers used are listed in Table 1. *E*. *coli* strains were grown in LB medium under aerobic conditions at 37°C and 200 rpm. Solid medium was prepared with the addition of 1.5% agar. *B*. *subtilis* cells were grown aerobically at 37°C and 200 rpm in NSMP media [24], nutrient sporulation medium with phosphate and were kept on Tryptose Blood Agar Base plates (TBAB, Difco). The antibiotic concentrations in use were: 12.5 μg/mL chloramphenicol and 100 μg/mL ampicillin for *E*. *coli*, and 5 μg/mL chloramphenicol for *B*. *subtilis*. Electrocompetent *E*. *coli* cells were prepared as described in [25] and the electroporation was conducted in a BioRad *E*. *coli* Pulser^™^ transformation apparatus. *B*. *subtilis* strains were grown to competence as described by Arwert and Venema [26], using at least one fresh plate with confluent growing bacteria for inoculation of 50 mL resuspension media.

**Table 1:** Bacterial strains, plasmids and primer.

**Bacteria**	**Relevant properties**	**Source**
*E*. *coli* XL1-Blue	*recA1*, *endA1*, *gyrA96*, *thi*, *hsdR17*, *supE44*, *relA1 (lac)*	Promega
*B*. *subtilis* 168A	*ΔmrpA ble*^*r*^	Bacillus Genetic Stock Center
*B*. *subtilis* ΔmrpA	ΔmrpA ble^r^	[17]
**Plasmids**		
pCW6	Cm^r^, shuttle vector	Claes von Wachenfeldt
pLch	nuoL fused with truncated *cccA*, his-added, Amp^r^	[27]
pAch	mrpA fused with truncated *cccA*, his-added, Amp^r^	[27]
pCWLch	nuoL fused with truncated *ccA*, his-added, Cm^r^	This work
pCWAch	mrpA fused with truncated *cccA*, his-added, Cm^r^	This work
**Primers**	**Primer sequence**	
MrpA_PCW6rev	5'-CGCCTTT*TCTAGA*TGCTTTTATC	
MrpA_PCW6fw	5'-CCGCTACTGT*CTGCAG*TCGTTTA	
NuoLrev	5'-ATGATTACGCC*AAGCTT*GAT	
Nuolfw	5'-ATT*GTCGA*CGGCGGCCTGTGAATG	
FL1-E144Q	5'P-CCTCGGCTGG*C*AAGGCGTGGGCCTG	
RL1-E144Q	5'P-CAGGCCCACGCCTT*G*CCAGCCGAGG	
FL2-K229A	5'P-GTGCGGTCGGT*GCA*TCTGCGCAGTTGC	
RL2-K229A	5'P-GCAACTGCGCAGA*TGC*ACCGACCGCAC	
FL3-H254A	5'P-CTCCGCGCTGATCG*CAG*CCGCAACCATGGTAACC	
RL3-H254A	5'P-GGTTACCATGGT*TGC*GGCTGCGATCAGCGCGGAG	
FL4-T257A	5'P-GATCCACGCCGCAG*CCA*TGGTAACCGC	
RL4-T257A	5'P-GCGGTTACCAT*GGC*TGCGGCGTGGATC	
FL5-M258I	5'P-CACGCCGCAACC*ATC*GTAACCGCGGG	
RL5-M258I	5'P-CCCGCGGTTAC*GAT*GGTTGCGGCGTG	
FL6-V259L	5'P-CGCAACCATG*CTAA*CCGCGGGTGTCTACC	
RL6-V259L	5'P-GGTAGACACCCGCGGT*TAG*CATGGTTGCG	
FA1-E113Q	5'P-GTTCTCTACATGTTCTGG*CAG*CTTACAAGCCTTTCATCC	
RA1-E113Q	5'P-GGATGAAAGGCTTGTAAG*CTG*CCAGAACATGTAGAGAAC	
FA2-K196A	5'P-CGGAAGGCATGGCTA*GCA*TCGGTGTATACGAAATC	
RA2-K196A	5'P-GATTTCGTATACACCGA*TGC*TAGCCATGCCTTCCG	
FA3-H221A	5'P-CCTGTCAGCGCTTATCTC*GCT*TCAGCTACTATGGTAAAAGC	
RA3-H221A	5'P-GCTTTTACCATAGTAGCTGA*AGC*GAGATAAGCGCTGACAGG	
FA4-T224A	5'P-GCGCTTATCTCCATTCAGCT*GCT*ATGGTAAAAGCCG	
RA4-T224A	5'P-CGGCTTTTACCATAGC*AGC*TGAATGGAGATAAGCGC	
FA5-M225I	5'P-GCGCTTATCTCCATTCAGCTACT*ATC*GTAAAAGCCGGC	
RA5-M225I	5'P-GCCGGCTTTTACGA*TAG*TAGCTGAATGGAGATAAGCGC	
FA6-V226L	5'P-CTCCATTCAGCTA*CTA*TGCTAAAAGCCGGCATTTATGTGATTGC	
RA6-V226L	5'P-GCAATCACATAAATGCCGGCTTTTAGCA*TAG*TAGCTGAATGGAG	
NuoLmut-seq	5'-CTTCTTCGCTTACACCAACC	
MrpA-seq1-forw	5'-CCTTTTATGTCTACTTATTGATG	

Restriction enzymes, ligase and DNA polymerase were purchased from Thermo Scientific and used according to manufacturer’s recommendations. The primers (Table 1) were synthesized by Invitrogen, Life Technologies. All PCR reactions were carried out in a BIORAD T100^™^ thermal cycler. DNA sequencing reactions were done using Big Dye^™^ (Applied Biosystems) and analyzed at the Biomolecular Resource Facility, Lund University.

### Construction of the pCWach and pCWlch plasmids

The two plasmids, pLCH and pACH [28], were used as templates for the amplification of *nuoLcytchis* and *mrpAcytchis* (the C-terminal end of NuoL and MrpA are fused to a cytochrome c domain, which has a his-tag). Primers in use are shown in Table 1. In the forward primer a unique PstI restriction site was introduced for the *mrpA* amplification and a SalI site was used in the case of *nuoL*. The reverse primers contained a XbaI site (MrpA) or a HindIII (NuoL) restriction site. The PCR conditions were 95°C initial denaturation for 3 min, followed by 30 cycles of 45 s at 95°C, 30 s annealing at 42°C, an elongation time of 2.5 min (nuoL) or 3 min (mrpA) at 72°C and ended by a final elongation time of 10 min at 72°C. For the PCR DreamTaq DNA polymerase was used. The PCR products were then digested with PstI/XbaI and HindIII/SalI respectively. The shuttle vector pCW6 was digested with the same enzymes and then ligated and transformed into *E*. *coli* JM109, by electroporation. The plasmids were named pCWach and pCWlch respectively. Those plasmids were then transformed into *B*. *subtilis ΔmrpA*, which were grown to competence using the method of Arwert and Venema [26]

### Site-directed mutagenesis

For the site directed mutagenesis of *nuoL* and *mrpA* the novel plasmids pCWlch and pCWach were used. Primers used for introducing the respective mutation are listed in Table 1. The mutagenesis was done with the Quick Change Site-Directed mutagenesis kit from Stratagene and was used according to the manufacturer’s instruction as well as using the Mega Primer method [29]. Positive clones were sequenced for the correct mutations and to exclude any extra mutations using the primers in Table 1.

### *B*. *subtilis* growth studies

The growth studies were conducted as described in Moparthi *et al*. [30]. The *B*. *subtilis* strains were grown in 35 mL batches in 250 mL baffled E-flasks with an attached sidearm consisting of 16 mm glass tube to facilitate OD measurements. The batches were incubated at 37°C at 200 rpm. The OD was measured every hour at 600 nm using a Biowave cell density meter C0800, which has an uncertainty of <± 0.05 A at 1A. The growth medium contained 8 g/L Nutrient Broth (NB, Difco, Becton Dickinson Co.), and 5 mL/L of a metal mixture (0.14 M CaCl_2_, 0.01 M MnCl_2_ and 0.2 M MgCl_2_). To retain the plasmid vectors chloramphenicol (5 μg/mL) was present. Since the expressed proteins harbor a cytochrome c domain, 1 μM FeCl_3_ was also included in the growth medium. 100 mM Tris-HCl was added to maintain constant pH of 7.4. According to the manufacturer, the NB contains 6.84 mM Na^+^ and 4.46 mM K^+^ when used as recommended. The NaCl concentrations indicated in each experiment always refers to the amount of NaCl added to the growth medium. The *B*. *subtilis* strains to be studied were taken from glycerol stock cultures at -80°C, and streaked on solid NB growth medium described above at pH 7.4 and with 1.5% agar, but no added NaCl. The plates were incubated at 37°C for 8 h, and the cells were then used immediately to inoculate the liquid cultures. Isopropyl-thio-β-D-galactoside (IPTG) for induction of protein expression from the P_spac_ promotor was present at 1 mM from the time of inoculation. After the last measurement the pH of the media was re-measured and a sample of the culture was re-streaked to verify no contamination. The growth studies (for each mutation) were repeated at least three times in independent experiments.

Growth curves of each strain were obtained by plotting the measured and averaged OD values against time. The *g* value was calculated using Eqs 1 and 2, where OD1 and OD2 are optical densities from the logarithmic growth phase, *t*1 and *t*2 the corresponding time points during logarithmic growth in minutes, and *k* is a growth constant.

$$k=\frac{\left(\text{ln}\left(OD2\right)-\text{ln}\left(OD1\right)\right)}{\left(t2-t1\right)}$$ (1)

$$g=\frac{\text{ln}2}{k}$$ (2)

### NMR-studies

The ^23^Na-NMR experiments were done as in [23]: first, biomass was obtained by growing *B*. *subtilis* cells in the same media as for growth studies, but without extra Na^+^ added. When the OD reached 0.5, 80 mM NaCl was added to challenge the cells with high sodium for about 1 h. The cells were then harvested, washed with Na^+-^free buffer (20 mM Tris-HCl, pH 7.4) and weighted, and resuspended to 0.32 g cells/ml buffer. An NMR sample consisted of 400 μl cell suspension, 10% D_2_O and 0.8 mM shift reagent Tm (DOTP) ^5^. The shift reagent, which does not penetrate the cell wall, was added to differentiate between intra and extracellular Na^+^. At least 512 scans were accumulated for each spectrum. Na^+^ concentrations were calculated from the integrated signal intensities and calibrated with a Na^+^ standard curve. The intracellular sodium signal was multiplied by 2.5, in order to compensate for the loss of 60% of the total signal intensity due to the extreme broadening of one of the components of the ^23^Na NMR signal from sodium inside the cell (see discussion in [23]).

## Results

### Pinpointing conserved amino acid residues specific for NuoL and MrpA

**Fig 1:**
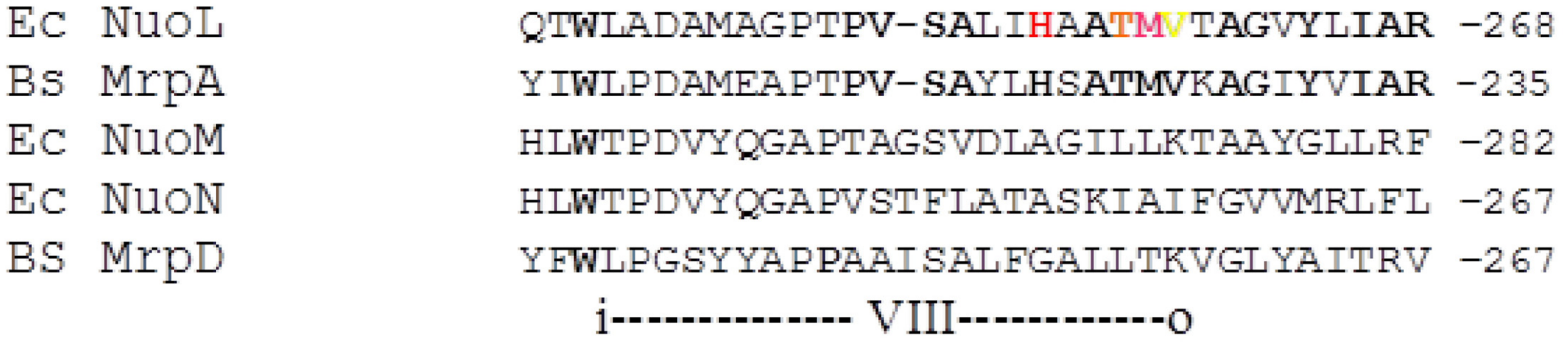
Sequence alignment of the Helix VIII area in relevant polypeptides from the entire protein family.

**Fig 2:**
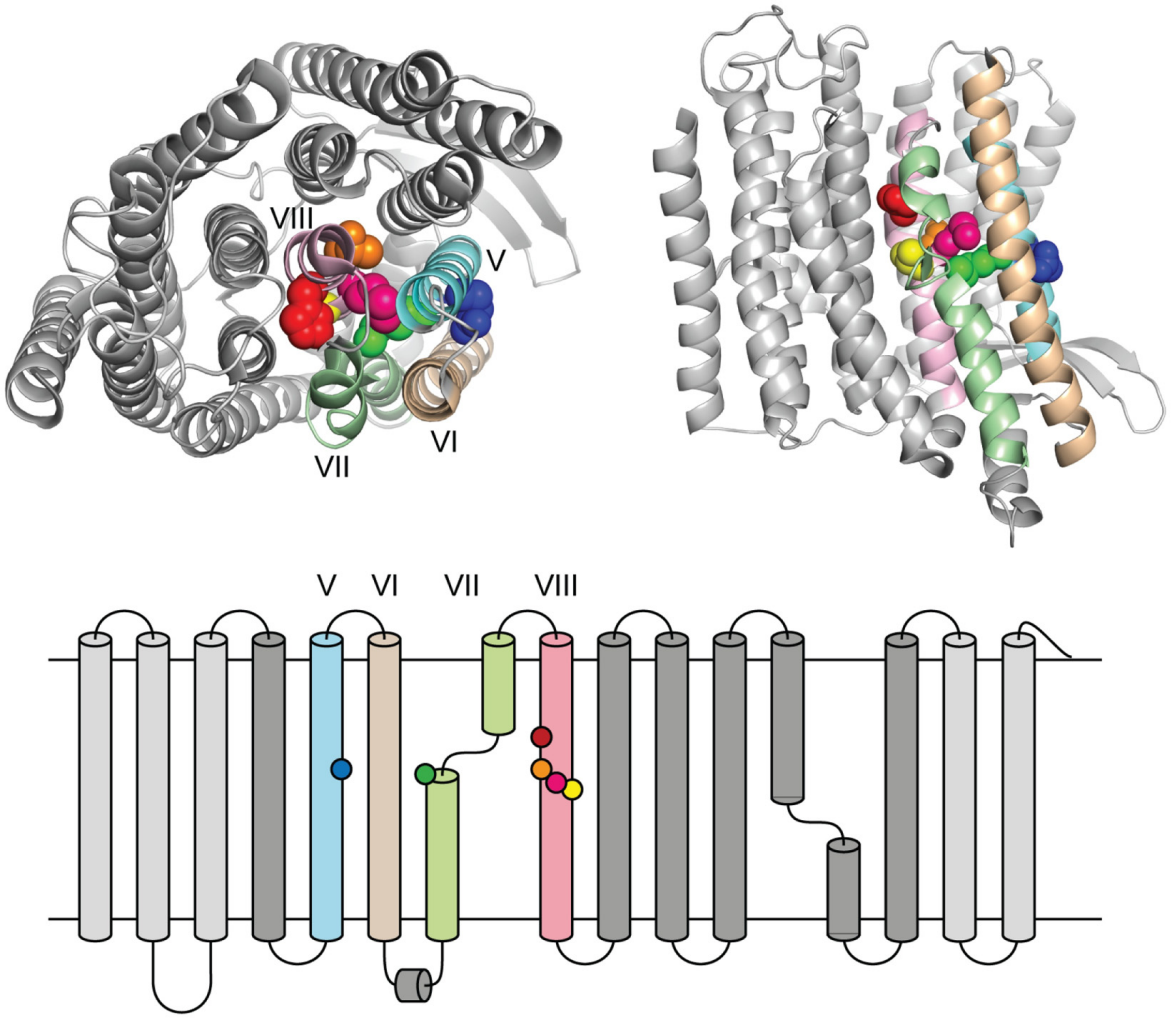
Location of helix VIII in the structure with mutated positions highlighted as in Fig 1 (based on a crystal structure of *E*. *coli* complex I (3RKO in PDB).

A large number of mutations in the complex I subunits NuoL [22], NuoM [31–33] and NuoN [18, 34] have been analyzed over the years, but the preferentially chosen positions for mutagenesis are those of fully conserved residues, as summarized in the supplementary material of [4] and in [35]. In this work we set out to look for positions that are conserved only in MrpA and NuoL (and not in the other three polypeptides, i.e. MrpD, NuoM and NuoN). One particular area of interest emerged after inspecting sequence alignments of the conserved TM helices [35]. One stretch of amino acids that is strongly conserved in only MrpA and NuoL was identified in helix VIII (Fig 1). Interestingly, helix VIII is located in the center of the antiporter-like subunits [4], adjacent to the half-helix structure and the fully conserved glutamate (NuoL E144) in helix V (Fig 2), a seemingly ideal location for a size exclusion function, expected of ion-specificity residues [36]. Matching conserved positions in both MrpA and NuoL were subsequently selected for mutagenesis (Fig 1). The positions chosen in helix VIII were for NuoL: H254A, T257A, M258I, V259L and for MrpA: H221A, T224A, M225I, V226L (see also Fig 2). In addition, two universally conserved positions in each polypeptide, that are known to severely affect complex I function [22, 37, 38], were mutated for testing in the model system, namely E144Q and K229A in NuoL, and the corresponding E113Q andn K196Ain MrpA. The latter mutant has not been described in the literature.

*E*. *coli* sequences are abbreviated Ec and *B*. *subtilis* sequences Bs. The conserved positions subjected to site-directed mutagenesis are indicated in colour in the *E*. *coli* NuoL sequence and shown with the same colours in the structure of NuoL in Fig 2.

The positions chosen for mutagenesis were, H254A, T257A, M258I, V259L for NuoL and the corresponding positions in MrpA, (H221A, T224A, M225I, V226L, see also Fig 1). In addition, two universally conserved positions that are known to severely affect complex I function [22] were mutated for testing in the model system; the position of E144Q is indicated by a blue dot K229A with by a green dot. In MrpA they correspond to E113Q and K196A.

### Complementation of the *ΔmrpA B*. *subtilis* strain with NuoL mutants

**Fig 3:**
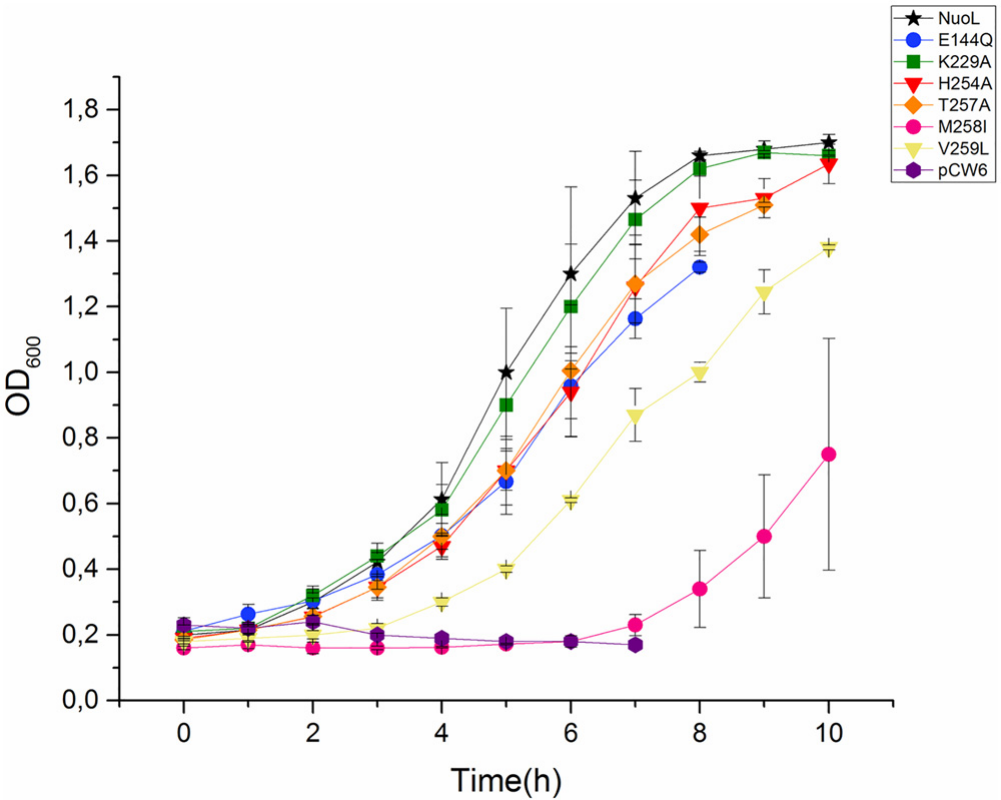
Growth properties of the *B*. *subtilis* deletion strains lacking MrpA and co-expressed with different mutations in subunit NuoL, at 80 mM NaCl.

The results of the complementation study of the different mutations (H254A, T257A, M258I, V259L, E144Q, and K229A) in the antiporter like subunit NuoL are shown in Fig 3. All six mutants were able to grow in 80 mM and pH 7.4. The strain with non-mutated NuoL grew as previously observed in Moparthi *et al* [39]. The lysine (K229A) mutant did not show any difference to the non-mutated version. Furthermore, the other mutants had a prolonged lag-phase and increased generation times. Valine (V259L) and methionine (M258I) mutants showed the most severe effects: valine (V259L) had a lag-phase of four hours and methionine (M258I) of six, after which they grew in a similar way as the control, see Table 2.

**Table 2:** Growth properties of *B*. *subtilis ΔmrpA* expressing the 6 different mutants.

	*B*. *subtilis ΔmrpA* at pH 7.4						
	Max OD	g (min)	lag (h)		Max OD	g (min)	lag (h)
80mM							
**MrpA**	1.82	75	1	**NuoL**	1.7	130	1
E113Q	1.84	69	0	E144Q	1.32	173	3
K196A	1.84	75	0	K229A	1.67	138	1
H221A	1.81	74	2	H254A	1.63	144	3
T224A	1.82	77	2	T257A	1.58	161	3
M225I	1.77	99	2	M258I	0.75	106	6
V226L	1.87	70	0	V259L	1.38	147	4
200mM							
**MrpA**	1,73	51	1				
E113Q	1,74	54	1				
K196A	1,76	74	1				
H221A	1,71	83	3				
T224A	1,68	75	2				
M225I	1,74	64	4				
V226L	1,75	65	1				

Lag = length of the lag-phase

g = generation time

Max OD = maximal OD after 8 or 10 hour

The growth was followed at pH 7.4. The non-mutated NuoL is represented by a black star. The mutations are seen as followed: E114Q mutation is shown as blue circle, K229A as green square, H254A as red triangle, T257A as orange diamond, M258I as pink circle, V259L as yellow triangle and the negative control as purple hexagon. The error bars shown in this figure represent the Standard Deviation (STD).

### Complementation of the *ΔmrpA B*. *subtilis* strain with MrpA mutants

**Fig 4:**
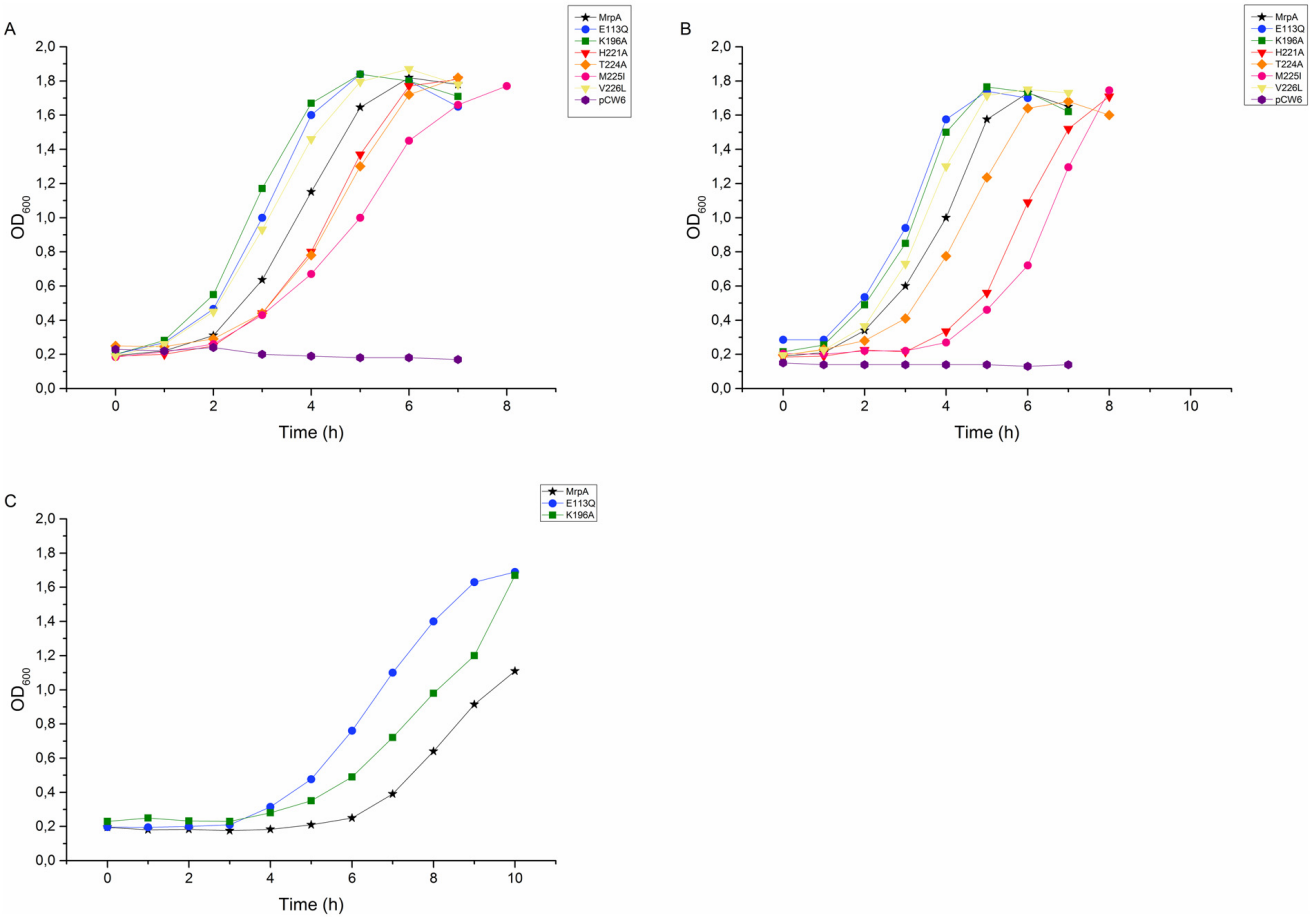
Growth properties of the *B*. *subtilis* deletion strains lacking MrpA and co-expressed with different mutations in subunit MrpA at 80 mM NaCl (A), 200 mM NaCl (B) and 800 mM NaCl (C) at pH 7.4.

All six mutants (H221A, T224A, M225I, V226L, E113Q, K196A), as well as the two controls (non-mutated *mrpA* and the empty vector pCW6), were expressed in trans in our *B*. *subtilis* model system. The results are shown in Fig 4. It is obvious from Fig 4A that all the different mutations were able to restore the growth in 80 mM NaCl, pH 7.4. Three of the mutants (H221A, M225I, and T224A) showed a longer lag-phase in comparison to the control, but similar generation times, which means that the shapes of growth curves are identical, but just shifted towards the longer time (Table 2, Fig 4A). Of those three, the methionine mutant M225I showed the most severe effect, with the longest generation time. Surprisingly, the other three mutants (E113Q, K196A, and V226L) had no lag-phase and started to grow immediately whereas the positive control had a slight lag-phase.

The differences between the growth of these mutants and the positive control were very small at 80 mM NaCl and seemed to become more visible in higher salt concentration. We measured the growth properties of the same mutants in 200 mM, and kept the same pH. The results are shown in Fig 4B. Under these circumstances the lag-phases were prolonged for all of the different samples, especially for the histidine (H221A) and the methionine mutants (M225I), where the lag-phase increased from two to almost four hours. We then increased the NaCl concentration to 800 mM to test the growth pattern of E113Q and K196A. Fig 4C shows the results. It is clearly visible that both mutants caused a prolonged lag-phase (in comparison to 80 and 200 mM NaCl), but still shorter than the lag-phase of the non-mutated proteins.

Whereas The different mutations are shown as follows: MrpA as black star, E113Q blue circle, K196A green square, H221A red triangle, T224A orange diamond, M225I pink circle, V226L yellow triangle and the negative control purple hexagon. The error bars shown in this figure represent the Standard Deviation.

### ^23^Na NMR measurements of intracellular sodium levels

**Fig 5:**
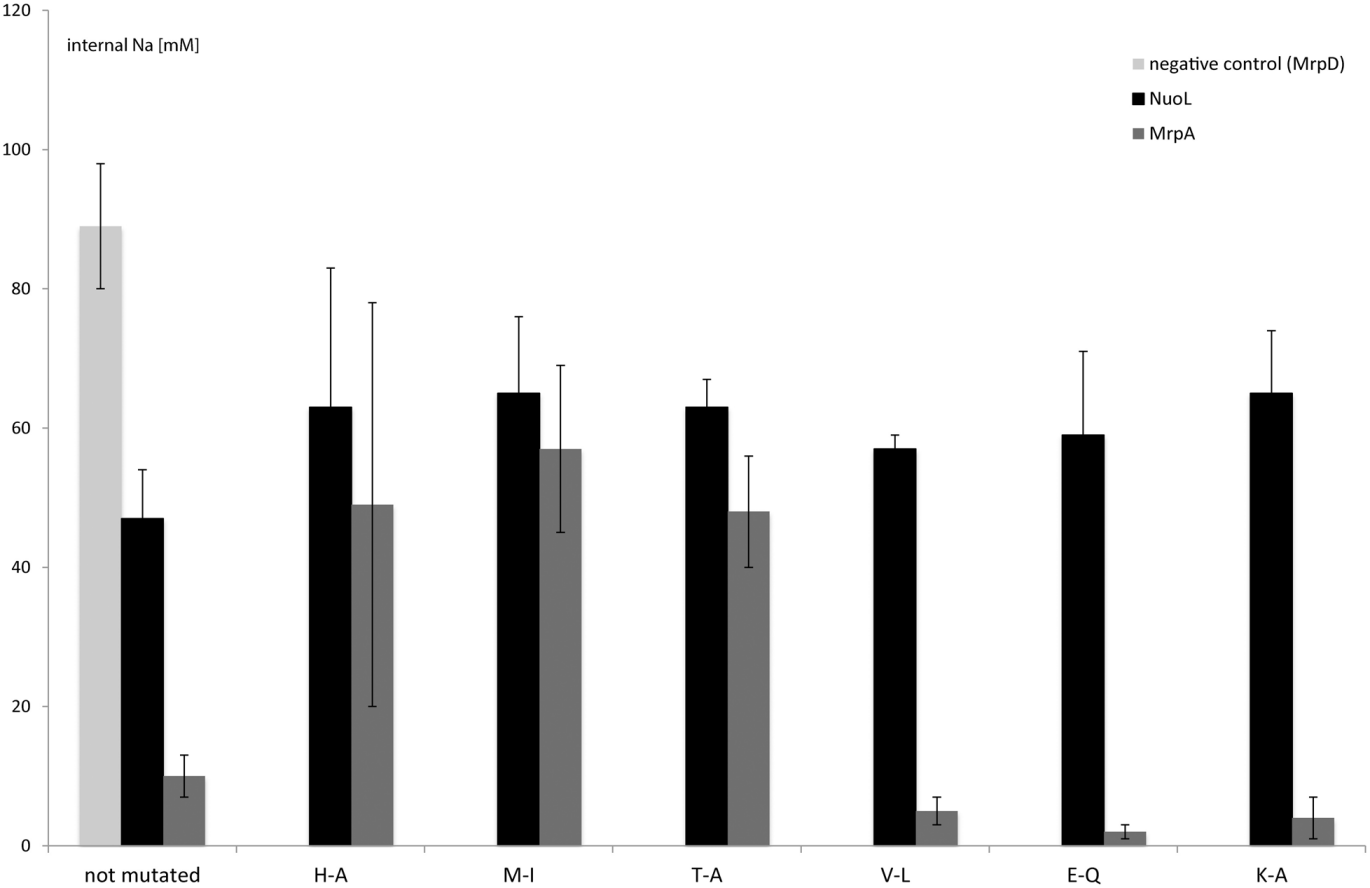
Comparison of the intracellular sodium concentrations of the different mutants and the designated positive controls as measured by ^23^Na NMR.

In order to show that the previously described effects of the mutations truly arose from sodium accumulation in the cells, we measured the sodium levels inside the intact cells by ^23^Na NMR. This method was previously used in our laboratory to measure sodium levels in the *B*. *subtilis* model system [23]. In the following description, comparisons are always made to the positive control (the cells with non-mutated subunit expressed *in trans*). Fig 5 shows the comparison between the intracellular sodium levels in mutants of the two different subunits. The three mutants in MrpA (E113Q, K196A, V226L), which showed a better growth than the non-mutated protein, contained also less sodium inside, whereas the other three mutants (H221A, M225I, T224A) had higher sodium levels in comparison. All in all methionine M225I showed the highest intracellular sodium levels.

It was obvious that NuoL-mutants (black bars) and the control had in general higher sodium levels than MrpA. We could detect a slight decrease in sodium-extrusion abilities in all mutants compared to the non-mutated NuoL. All six mutants had very similar amounts of intracellular sodium, whereas valine mutant had the lowest and lysine and methionine the highest of them.

The black bars represent the NuoL mutants, the dark grey the MrpA and the light grey the negative control. The cells were grown in sodium-free medium until OD 0.5, then 80 mM NaCl was added. After 1–2 h in high Na, the cells were spun down, washed in Na^+^-free buffer and resuspended to 0.32 g cell / ml in the buffer. The Na^+^ concentrations were measured immediately by ^23^Na-NMR. Each mutant was measured at least four independent times and the error bars stated in this figure represent SD’s.

## Discussion

A sequence motif that is conserved only in MrpA-like polypeptides but not in MrpD-like polypeptides was identified in TM helix VIII. This motif has a promising position in the solved structure being close to the broken-helix VII, which might be important for function and even responsible for ion specificity. Thus, we chose a number of conserved positions in helix VIII to mutate and study their effects.

This work demonstrated that we were able to pinpoint interesting mutations and characterize their roles in the antiporter subunits of Mrp and the antiporter-like subunits of complex I. This was achieved by a combination of two different methods, our *B*. *subtilis* growth studies and ^23^Na NMR measurements of intracellular sodium levels, which complement each other remarkably well, proving that the observed effects were indeed due to the cells’ inability to extrude excess sodium.

In a previous study it was concluded that MrpA and MrpD are single ion channels, where MrpA is responsible for Na^+^ translocation and MrpD for H^+^ and together form an antiporter. Furthermore it was shown that the corresponding complex I subunits NuoL and NuoN could be grouped with either MrpA or MrpD. Consequently these proteins showed not only primary sequence similarity, but also an existing functional similarity [17].

But in this work we showed that although MrpA and NuoL seem to be very similar in sequence they may differ on the functional level. Especially mutating the valine (V259L, V226L) residue gave drastically different results in the two subunits. In NuoL the valine mutation to leucine (V259L) slowed the growth significantly (the second longest lag-phase), whereas in MrpA (V226L) the growth was improved in comparison to the non-mutated version. This result was even more pronounced in ^23^Na NMR measurements, where the mutation of valine to leucine improved the sodium extrusion abilities in MrpA compared to the non-mutated protein, whereas the same mutation in NuoL had an opposite effect. Mutating the histidine residue to alanine showed next to the methionine residue a prolonged lag-phase in MrpA, whereas in NuoL it was nearly the same as the control. The only mutation, which showed a severe effect in both NuoL and MrpA was the methionine mutation. Therefore we conclude that this amino acid plays an important role in both proteins.

Interestingly, we could detect improved growths and increase in sodium-extrusion abilities in three of the mutants of MrpA, two of which were expected to be detrimental to the function based on previous studies. One of those mutants in MrpA E113Q has been previously described in Kajiyama *et al*. [38] and was shown not to be able to grow in high NaCl concentrations (0.2 M). We were able to grow this mutant in 80 mM, 200 mM, and 800 mM (Fig 4A, 4B and 4C) and also the ^23^Na NMR showed that the mutant was able to extrude sodium to an extent comparable to the wildtype. The corresponding mutations in NuoL (E144Q), and also K229A, have been previously reported to have a low proton pumping activity as well as oxidoreductase activity [22], whereas in our experimental setup we could only see a slight difference between the mutant and the non-mutated NuoL.

Since our experiments were not performed in complex I context, we did not measure either proton pumping or oxidoreductase activity. However we think that our phenotypes are strictly sodium-dependent and not based on an altered proton gradient, because cells that are not challenged with high salt concentration grew equally well compared to the positive control. However we cannot exclude that the mutations to some degree compromise the stability of the protein, but a more likely explanation for the presented results is that they simply reflect actual effects on sodium transport activities in the mutated proteins.

As previously reported by Nakamaru-Ogiso *et al*. [22], the mutations E144Q and K229A in NuoL severely reduced complex I activity. In our model system, however, those mutations had almost no effect on the cell growth, and the intracellular sodium levels were comparable to other mutants. Even more striking was the effect of corresponding mutations in MrpA subunit: not only was the function not lost by mutating those seemingly crucial residues, but in fact the cells expressing those mutants performed better in comparison to the positive control. It is obvious that the polypeptides behave differently in different contexts (in *E*. *coli* membrane vesicles containing complex I versus *B*. *subtilis* whole cells grown in high salt medium). This means the polypeptides may have more than one role, therefore including our approach into complex I studies can in fact help to retrieve more information about the function of Nuo subunits.

Histidine 254 in NuoL was suggested to have a role in complex I proton pumping mechanism, as it is a part of the long chain of charged residues running parallel to the membrane through the whole membrane domain of complex I [40, 41]. Our results showed that it behaved similarly to the other two residues from this long chain (E144 and K229).

Our data present effects of new mutations in complex I and the Mrp antiporter studied by a combination of two different methods. The results may help elucidate the mechanism of ion translocation by complex I and the evolutionary relationship between complex I and the Mrp antiporter.

## References

[1] Efremov RG, Baradaran R, Sazanov LA. The architecture of respiratory complex I. Nature. 2010;465(7297):441–5. 10.1038/nature09066 . 20505720

[2] Sazanov LA, Hinchliffe P. Structure of the hydrophilic domain of respiratory complex I from Thermus thermophilus. Science. 2006;311(5766):1430–6. 10.1126/science.1123809 . 16469879

[3] Hunte C, Zickermann V, Brandt U. Functional modules and structural basis of conformational coupling in mitochondrial complex I. Science. 2010;329(5990):448–51. Epub 2010/07/03. science.1191046 [pii] 10.1126/science.1191046 . 20595580

[4] Efremov RG, Sazanov LA. Structure of the membrane domain of respiratory complex I. Nature. 2011;476(7361):414–20. 10.1038/nature10330 . 21822288

[5] Baradaran R, Berrisford JM, Minhas GS, Sazanov LA. Crystal structure of the entire respiratory complex I. Nature. 2013 10.1038/nature11871 PMC3672946 23417064

[6] Friedrich T. The NADH:ubiquinone oxidoreductase (complex I) from Escherichia coli. Biochimica Et Biophysica Acta Bioenergetics. 1998;1364(2):134–46. 10.1016/s0005-2728(98)00024-3 9593861

[7] Yagi T, Yano T, Di Bernardo S, Matsuno-Yagi A. Procaryotic complex I (NDH-1), an overview. Bba-Bioenergetics. 1998;1364(2):125–33. ISI:000073897100005. 9593856 10.1016/s0005-2728(98)00023-1

[8] Brandt U. Energy converting NADH: Quinone oxidoreductase (Complex I). Annu Rev Biochem. 2006;75:69–92. 10.1146/annurev.biochem.75.103004.142539 ISI:000239807600004. 16756485

[9] Galkin A, Grivennikova VG., Vinogradov AD. H+/e- stoichiometry in NADH-quinone reductase reactions catalyzed by bovine heart submitochondrial particles. Febs Lett. 1999;451(2):157–61. 10371157 10.1016/s0014-5793(99)00575-x

[10] Ripple MO, Kim N, Springett R. Mammalian complex I pumps 4 protons per 2 electrons at high and physiological proton motive force in living cells. J Biol Chem. 2013;288(8):5374–80. 10.1074/jbc.M112.438945 23306206 PMC3581419

[11] Wikstrom M, Hummer G. Stoichiometry of proton translocation by respiratory complex I and its mechanistic implications. Proc Natl Acad Sci U S A. 2012;109(12):4431–6. 10.1073/pnas.1120949109 22392981 PMC3311377

[12] Fearnley IM, Walker JE. Conservation of sequences of subunits of mitochondrial complex I and their relationships with other proteins. Biochim Biophys Acta. 1992;1140(2):105–34. 1445936 10.1016/0005-2728(92)90001-i

[13] Hamamoto T, Hashimoto M, Hino M, Kitada M, Seto Y, Kudo T, et al Characterization of a gene responsible for the Na+/H+ antiporter system of alkalophilic Bacillus species strain C-125. Molecular microbiology. 1994;14(5):939–46. Epub 1994/12/01. . 7715455 10.1111/j.1365-2958.1994.tb01329.x

[14] Swartz TH, Ikewada S, Ishikawa O, Ito M, Krulwich TA. The Mrp system: a giant among monovalent cation/proton antiporters? Extremophiles: life under extreme conditions. 2005;9(5):345–54. 10.1007/s00792-005-0451-6 . 15980940

[15] Mathiesen C, Hägerhäll C. Transmembrane topology of the NuoL, M and N subunits of NADH: quinone oxidoreductase and their homologues among membrane-bound hydrogenases and bona fide antiporters. Biochim Biophys Acta. 2002;1556(2–3):121–32. ISI:000179691200006. 12460669 10.1016/s0005-2728(02)00343-2

[16] Moparthi VK, Kumar B, Al-Eryani Y, Sperling E, Gorecki K, Drakenberg T, et al Functional role of the MrpA- and MrpD-homologous protein subunits in enzyme complexes evolutionary related to respiratory chain complex I. Biochim Biophys Acta. 2014;1837(1):178–85. 10.1016/j.bbabio.2013.09.012 . 24095649

[17] Moparthi VK, Kumar B, Mathiesen C, Hagerhall C. Homologous protein subunits from Escherichia coli NADH:quinone oxidoreductase can functionally replace MrpA and MrpD in Bacillus subtilis. Biochim Biophys Acta. 2011;1807(4):427–36. 10.1016/j.bbabio.2011.01.005 . 21236240

[18] Michel J, DeLeon-Rangel J, Zhu ST, Van Ree K, Vik SB. Mutagenesis of the L, M, and N Subunits of Complex I from Escherichia coli Indicates a Common Role in Function. Plos One. 2011;6(2). ARTN e17420 10.1371/journal.pone.0017420 WOS:000287931400073. 21387012 PMC3046159

[19] Verkhovskaya M, Bloch DA. Energy-converting respiratory Complex I: on the way to the molecular mechanism of the proton pump. Int J Biochem Cell Biol. 2013;45(2):491–511. Epub 2012/09/18. S1357-2725(12)00302-0 [pii] 10.1016/j.biocel.2012.08.024 . 22982742

[20] Morino M, Natsui S, Ono T, Swartz TH, Krulwich TA, Ito M. Single site mutations in the hetero-oligomeric Mrp antiporter from alkaliphilic Bacillus pseudofirmus OF4 that affect Na+/H+ antiport activity, sodium exclusion, individual Mrp protein levels, or Mrp complex formation. J Biol Chem. 2010;285(40):30942–50. 10.1074/jbc.M110.118661 20624916 PMC2945585

[21] Forrest LR, Kramer R, Ziegler C. The structural basis of secondary active transport mechanisms. Biochim Biophys Acta. 2011;1807(2):167–88. 10.1016/j.bbabio.2010.10.014 . 21029721

[22] Nakamaru-Ogiso E, Kao MC, Chen H, Sinha SC, Yagi T, Ohnishi T. The Membrane Subunit NuoL(ND5) Is Involved in the Indirect Proton Pumping Mechanism of Escherichia coli Complex I. Journal of Biological Chemistry. 2010;285(50):39070–8. 10.1074/jbc.M110.157826 WOS:000284941300037. 20826797 PMC2998099

[23] Gorecki K, Hagerhall C, Drakenberg T. The Na+ transport in gram-positive bacteria defect in the Mrp antiporter complex measured with 23Na nuclear magnetic resonance. Analytical biochemistry. 2014;445:80–6. 10.1016/j.ab.2013.10.003 . 24139955

[24] Fortnagel P, Freese E. Analysis of Sporulation Mutants .2. Mutants Blocked in Citric Acid Cycle. J Bacteriol. 1968;95(4):1431–8. ISI:A1968A987100032. 4967197 10.1128/jb.95.4.1431-1438.1968 PMC315104

[25] Sambrook S, Fritsch EF, Maniatis T. Molecular cloning. A laboratory manual. 2nd ed Cold Spring Harbor, NY: Cold Spring Harbor Laboratory; 1989.

[26] Arwert F, Venema G. Transformation in Bacillus-Subtilis—Fate of Newly Introduced Transforming DNA. Molecular & General Genetics. 1973;123(2):185–98. ISI:A1973Q135800007. 4199220 10.1007/BF00267334

[27] Gustavsson T, Trane M, Moparthi VK, Miklovyte E, Moparthi L, Gorecki K, et al A cytochrome c fusion protein domain for convenient detection, quantification, and enhanced production of membrane proteins in Escherichia coli—expression and characterization of cytochrome-tagged Complex I subunits. Protein Sci. 2010;19(8):1445–60. 10.1002/pro.424 20509166 PMC2923498

[28] Gustavsson T, Trane M, Moparthi VK, Miklovyte E, Moparthi L, Gorecki K, et al A cytochrome c fusion protein domain for convenient detection, quantification, and enhanced production of membrane proteins in Escherichia coli-Expression and characterization of cytochrome-tagged Complex I subunits. Protein Sci. 2010;19(8):1445–60. 10.1002/Pro.424 ISI:000280481300001. 20509166 PMC2923498

[29] Tseng WC, Lin JW, Wei TY, Fang TY. A novel megaprimed and ligase-free, PCR-based, site-directed mutagenesis method. Analytical biochemistry. 2008;375(2):376–8. 10.1016/j.ab.2007.12.013 . 18198125

[30] Moparthi VK, Kumar B, Mathiesen C, Hagerhall C. Homologous protein subunits from Escherichia coli NADH:quinone oxidoreductase can functionally replace MrpA and MrpD in Bacillus subtilis. Biochim Biophys Acta Bioenergetics. 2011;1807:427–36. 10.1016/j.bbabio.2011.01.005. 21236240

[31] Torres-Bacete J, Nakamaru-Ogiso E, Matsuno-Yagi A, Yagi T. Characterization of the NuoM (ND4) subunit in Escherichia coli NDH-1—Conserved charged residues essential for energy-coupled activities. J Biol Chem. 2007;282(51):36914–22. ISI:000251646000017. 17977822 10.1074/jbc.M707855200

[32] Torres-Bacete J, Sinha PK, Castro-Guerrero N, Matsuno-Yagi A, Yagi T. Features of Subunit NuoM (ND4) in Escherichia coli NDH-1 topology and implications of conserved Glu(144) for coupling site 1. J Biol Chem. 2009;284(48):33062–9. ISI:000272028500009. 10.1074/jbc.M109.059154 19815558 PMC2785147

[33] Euro L, Belevich G, Verkhovsky MI, Wikstrom M, Verkhovskaya M. Conserved lysine residues of the membrane subunit NuoM are involved in energy conversion by the proton-pumping NADH: ubiquinone oxidoreductase (Complex I). Biochim Biophys Acta. 2008;1777(9):1166–72. ISI:000259287200011. 10.1016/j.bbabio.2008.06.001 18590697

[34] Amarneh B, Vik SB. Mutagenesis of subunit N of the Escherichia coli complex I. Identification of the initiation codon and the sensitivity of mutants to decylubiquinone. Biochemistry. 2003;42(17):4800–8. 10.1021/bi0340346 . 12718520

[35] Moparthi VK, Hägerhäll C. Recruitment of the antiporter module—a key event in Complex I evolution In: Sazanov LA, editor. A structural perspective on complex I: Springer; 2011.

[36] Hunte C, Screpanti E, Venturi M, Rimon A, Padan E, Michel H. Structure of a Na+/H+ antiporter and insights into mechanism of action and regulation by pH. Nature. 2005;435(7046):1197–202. 10.1038/nature03692 . 15988517

[37] Kosono S, Kajiyama Y, Kawasaki S, Yoshinaka T, Haga K, Kudo T. Functional involvement of membrane-embedded and conserved acidic residues in the ShaA subunit of the multigene-encoded Na+/H+ antiporter in Bacillus subtilis. Biochim Biophys Acta. 2006;1758(5):627–35. 10.1016/j.bbamem.2006.04.012 . 16730649

[38] Kajiyama Y, Otagiri M, Sekiguchi J, Kudo T, Kosono S. The MrpA, MrpB and MrpD subunits of the Mrp antiporter complex in Bacillus subtilis contain membrane-embedded and essential acidic residues. Microbiology. 2009;155(Pt 7):2137–47. 10.1099/mic.0.025205-0 . 19389778

[39] Moparthi VK, Kumar B, Mathiesen C, Hagerhall C. Homologous protein subunits from Escherichia coli NADH:quinone oxidoreductase can functionally replace MrpA and MrpD in Bacillus subtilis. Biochim Biophys Acta. 2011;1807(4):427–36. Epub 2011/01/18. 10.1016/j.bbabio.2011.01.005 . 21236240

[40] Hirst J. Mitochondrial complex I. Annu Rev Biochem. 2013;82:551–75. 10.1146/annurev-biochem-070511-103700 . 23527692

[41] Sazanov LA. The mechanism of coupling between electron transfer and proton translocation in respiratory complex I. J Bioenerg Biomembr. 2014;46(4):247–53. 10.1007/s10863-014-9554-z . 24943718

